# Indoor and Outdoor Cultures of *Gracilaria chilensis*: Determination of Biomass Growth and Molecular Markers for Biomass Quality Evaluation

**DOI:** 10.3390/plants12061340

**Published:** 2023-03-16

**Authors:** Sofía Caroca-Valencia, Jorge Rivas, Matías Araya, Alejandra Núñez, Florentina Piña, Fernanda Toro-Mellado, Loretto Contreras-Porcia

**Affiliations:** 1 Departamento de Ecología y Biodiversidad, Facultad de Ciencias de la Vida, Universidad Andres Bello, República 440, Santiago 8370251, Chile; 2 Centro de Investigación Marina Quintay (CIMARQ), Facultad de Ciencias de la Vida, Universidad Andres Bello, Valparaíso 2531015, Chile; 3 Center of Applied Ecology and Sustainability (CAPES), Santiago 8331150, Chile; 4 Instituto Milenio en Socio-Ecología Costera (SECOS), Santiago 8370251, Chile; 5 Programa de Doctorado en Biotecnología, Facultad de Ciencias de la Vida, Universidad Andres Bello, Santiago 8370251, Chile

**Keywords:** aquaculture, antioxidant capacity, marine food, seaweed production, seaweed culture, sustainability

## Abstract

Taking into consideration climate change scenarios, marine contamination, and a constantly expanding world population, seaweed aquaculture has become an important option for the large-scale production of high-quality biomass. Due to existing biological knowledge of *Gracilaria chilensis*, several cultivation strategies have been established for obtaining diverse biomolecules (lipids, fatty acids, pigments, among others) with nutraceutical properties. In this research, indoor and outdoor cultivation methodologies were applied to generate high biomass of *G. chilensis* with positive quality for productive purposes, where the quality was determined according to the concentrations of lipoperoxides and phenolic compounds and the total antioxidant capacity (TAC). The results showed that *G. chilensis* cultures, which were fertilized for three weeks with Basfoliar^®^ Aktiv (BF) at concentrations of 0.05–1% *v*/*v*, obtained high biomass (1–1.3 kg m^–2^) and DGR (0.35–4.66% d^–1^), low lipoperoxides (0.5–2.8 µmol g^–1^ DT), and high phenolic compounds (0.4–0.92 µ eq. GA g^–1^ FT) and TAC (5–7.5 nmol eq. TROLOX g^–1^ FT) as compared with other culture media. Lower stress was determined under indoor cultures, due to the operative control of diverse physicochemical stressor parameters (T°, light intensity, photoperiod, among others). Therefore, the cultures developed allow scaling the biomass in productive terms and are suitable for obtaining compounds of interest.

## 1. Introduction

The search to satisfy the demand for food and feed and to address the challenges of an ever-expanding population, taking into consideration climate change scenarios, has resulted in the need to achieve high-quality biomass production from nontraditional sources such as large-scale seaweed aquaculture. The Food and Agriculture Organization of the United Nations (FAO) 2020 report entitled “The State of World Fisheries and Aquaculture” indicated that the world production of seaweed was 32.4 million tons, of which 97.1% came from cultures [1]. This report mentioned the importance of these organisms for research, for example, in the areas of medicine, cosmetics, water treatment, food industry, and biofuels [1]. In this context, seaweeds stand out for their properties and benefits towards human health and the development of functional aliments [2,3].

Red algae, particularly those of the genus *Gracilaria*, have been described as a complex lipid composition of glycerolipids, omega-3 and omega-6, and oxylipin-like fatty acids [4,5]. In the case of *Gracilaria chilensis* (known as Pelillo), essential fatty acids, such as polyunsaturated fatty acids (PUFA) have been described [6,7,8,9]. Recently, our laboratory reported that Gracilex^®^, an oleoresin derived from *G. chilensis*, was enriched in palmitic acid, arachidonic acid, oleic acid, and lipophilic antioxidants such as tocopherols and β–carotene [9]. Gracilex^®^ can reduce metabolic alterations (increases in basal glucose and insulin levels) in mice fed with a high-fat diet [9]. Thus, to obtain those compounds, from a commercial perspective, it is necessary to have large-scale and high-quality biomass production [10]. 

*G. chilensis* is the most important cultivated red seaweed species in Chile [11]. Part of the seaweed that is harvested is destined to produce agar, a phycocolloid used in the pharmaceutical and food industries, which is exported principally in the form of dried raw material [11]. The locations of culture centers for this species range from Antofagasta (23° S) to Coyhaique (45° S), but the largest number of culture zones (88%) and volume of production (77%) are concentrated in the Los Lagos Region (41° S). Large-scale cultivation methodologies of *G. chilensis* are possible due to the basic knowledge of key biological aspects, such as propagation methods and ecophysiological responses under diverse culture conditions. Today, *G. chilensis* cultivation is mainly by vegetative propagation of the cultivars in marine farms [12].

Among the current methodologies for seaweed cultivation, indoor and outdoor cultivation systems control biomass growth and quality without negative externalities on the environment [13,14,15]. According to the authors of [14], the benefits of indoor and outdoor cultivation systems include possible fertilization, i.e., supplying nutrients in adequate amounts to obtain positive cell growth [16,17], as well as the removal of contaminants from the biomass, such as heavy metals, ensuring a high quality [15]. One of the most commonly used media is Von Stosch (VS), due to its contribution of nitrogen, phosphorus, trace metals (such as manganese and iron, among others), and vitamins such as B_12_, B_7_, and B_1_, which have been shown to be effective in laboratory-scale indoor cultures for the initial development stages of red algae species [18,19]. In addition, Basfoliar^®^ Aktiv (BF) is a concentrate of macro- and micronutrients from seaweeds which has been beneficial for the initial growth of *G. chilensis* in indoor cultures [14,20]. 

Methods to assess the quality of vegetal biomass, in terms of biomolecules of interest, use markers of oxidative stress, a condition characterized by a net increase in reactive oxygen species (ROS) that results from insufficient scavenging of antioxidant defenses under several stressors [21]. The oxidative stress condition triggers physiological alterations that can impact the production of biomolecules of interest [22]. Among the most widely used markers for detecting oxidative damage and biomass quality are lipid peroxidation and quantification of antioxidant compounds [22], such as phenolic compounds and total antioxidant capacity (TAC) of the biomass [23,24]. Therefore, relating biomass growth to quality can result in suitable biomasses for the extraction and production of compounds for nutritional purposes. We hypothesized that indoor and outdoor cultures of *G. chilensis* previously fertilized with different culture media will generate high biomass with adequate quality. The results will provide added value to controlled cultures of this species.

## 2. Results

### 2.1. Biomass and Daily Growth Rate (DGR)

**Figure 1:**
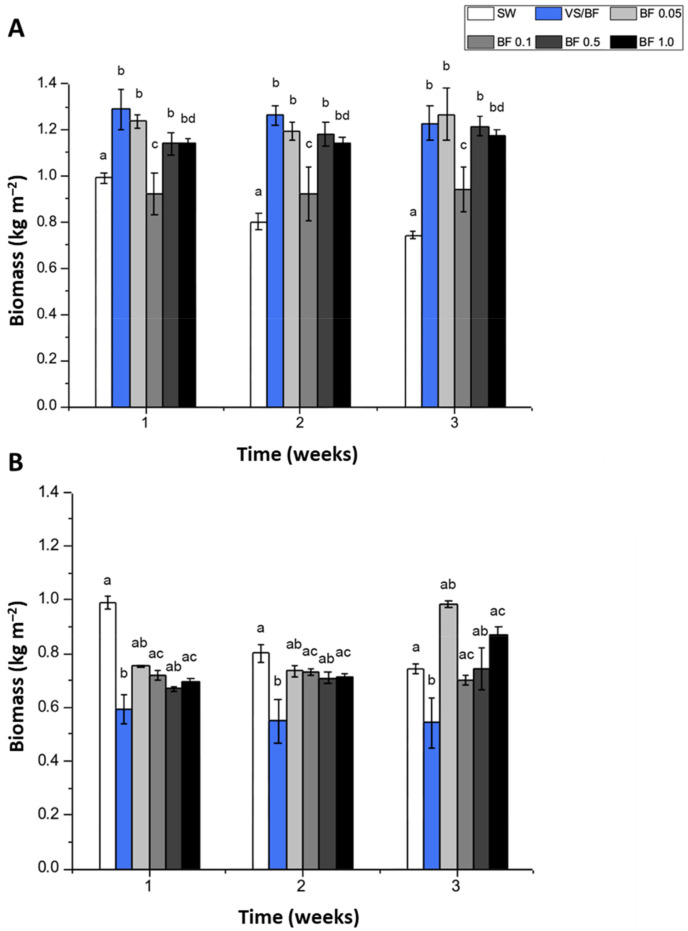
Biomass (kg m^–2^) of *G. chilensis* obtained under (**A**) indoor and (**B**) outdoor cultivation systems for each treatment evaluated (seawater (SW) treatment or control), Von Stosch/Basfoliar^®^ Aktiv (VS/BF) treatment, and Basfoliar^®^ Aktiv (0.05%, 0.1%, 0.5%, and 1% *v*/*v*) treatments) during the three weeks of the experimental period. Values are the mean ± SD of three replicates. The letters above the bar plots indicate the results of Dunn’s tests; means with the same letter are not significantly different at *p* ≥ 0.05.

The biomass (kg m^–2^) in the indoor cultivation system using seawater (control or SW treatment) did not increase during the experimental period and showed a tendency to decrease during the third experimental week (Figure 1A). Contrarily, the biomasses exposed to all treatments increased significantly in the third week of cultivation (in all cases *p* < 0.05) (ca. 0.9–1.3 kg m^–2^) with an increase between 26 to 70% (Figure 1A). The experimental factor that proved to be influential on biomass, according to the Kruskal–Wallis test, was the culture media used (*p* < 0.001). In the case of the biomass cultivated in an outdoor system, SW treatment showed an increase at the end of the first week of the experimental period, but a clear decrease during the remaining weeks of the experimental period (Figure 1B). A similar outcome was obtained with VS/BF treatment (Figure 1B). Regarding the BF media at different concentrations, a decrease in biomass was observed in the first and second week with respect to the control treatment, but an increase was obtained to BF 0.05 (32.3%) and BF 1.0 (17.2%) to the third week (Figure 1B). The medium that showed the highest increase in biomass at the end of the experimental period was BF 0.05%, which reached 1 kg m^–2^. 

**Figure 2:**
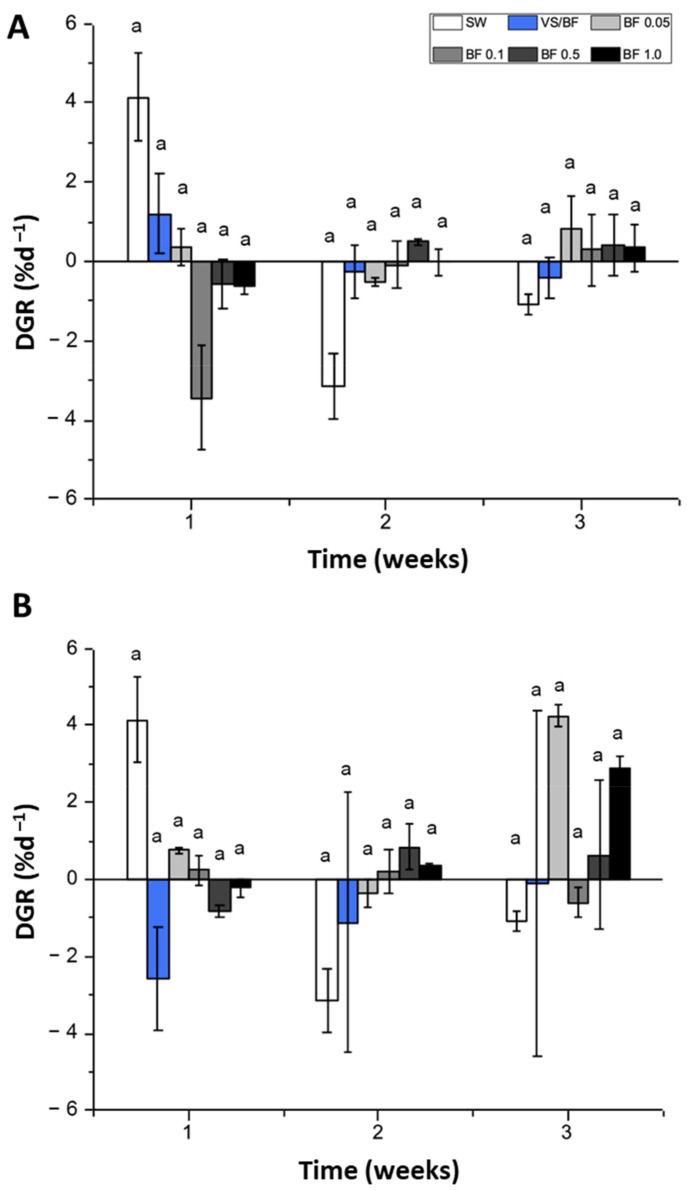
DGR (% d^–1^) of *G. chilensis* obtained under (**A**) indoor and (**B**) outdoor cultivation systems for each treatment evaluated (seawater (SW) treatment or control, Von Stosch/Basfoliar^®^ Aktiv (VS/BF), and Basfoliar^®^ Aktiv (0.05%, 0.1%, 0.5%, and 1% *v*/*v*) treatments) during the three weeks of the experimental period. Values are the mean ± SD of three replicates. The letters above the bar plots indicate the results of Dunn´s tests; means with the same letter are not significantly different at *p* ≥ 0.05.

For the indoor cultivation system, the daily growth rate (DGR) (% d^–1^) of *G. chilensis* in the control, i.e., seawater treatment (SW), showed a decrease during the second and third weeks of the experimental period, which also occurred with the VS/BF treatment (Figure 2A). In the case of BF 0.05%, the first week of the experimental period showed growth, with a decrease during the second week, and then a positive increase during the third week (Figure 2A). For the BF 0.1%, 0.5%, and 1% treatments, there was a decrease during the first week of experimental period; this behavior was maintained during the second week, except for the BF 0.5% treatment (Figure 2A). During the third week of experimental period, the BF 0.05%, 0.1%, 0.5%, and 1% treatments showed positive growth rates, with values of 0.35, 0.50, and 0.83% d^–1^, respectively. According to the Kruskal–Wallis test, no significant incidences of treatments, experimental time, or the combination of both factors on the DGR were found (*p* = 0.225). In the case of the outdoor cultivation system, *G. chilensis* showed a positive value for the SW, BF 0.05%, and BF 0.1% treatments, which changed in the second week of the experimental week (Figure 2B). The treatment with BF 0.05% medium obtained the highest DGR with a value of 4.66 ± 0.23% d^–1^ at the end of the experimental period (Figure 2B). For this parameter, no statistical significance was found between the data, according to the Kruskal–Wallis test (*p* > 0.05). 

### 2.2. Molecular Markers 

**Figure 3:**
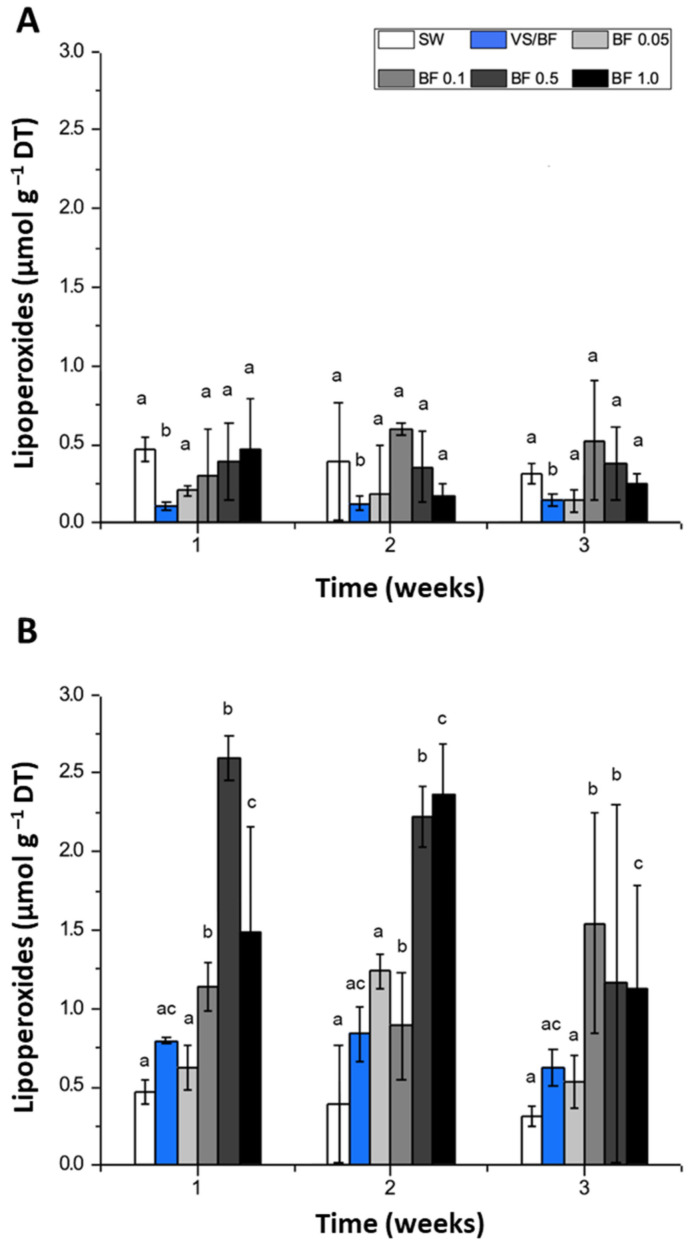
Lipoperoxide concentration (µmol g^–1^ DT, dry tissue) in *G. chilensis* obtained under (**A**) indoor and (**B**) outdoor cultivation systems for each treatment evaluated (seawater (SW) control, Von Stosch/Basfoliar^®^ Aktiv (VS/BF) treatment, and Basfoliar^®^ Aktiv (0.05%, 0.1%, 0.5%, and 1% *v*/*v*) treatments) during the three weeks of the experimental period. Values are the mean ± SD of three replicates. The letters above the bar plots indicate the results of Dunn´s tests; means with the same letter are not significantly different at *p* ≥ 0.05.

In the indoor cultivation system, the concentration of lipoperoxides determined in the tissue of *G. chilensis* from the control seawater treatment (SW) remained stable during the entire experimental period (Figure 3A) (0.4–0.5 µmol g^–1^ DT). Similar patterns were registered using the VS/BF treatment and the BF concentration treatments. No differences were registered among the treatments (*p* > 0.01). In the outdoor cultivation system, the concentration of lipoperoxides from the control treatment remained stable during the entire experimental period (Figure 3B) (0.4–0.5 µmol g^–1^ DT). A similar pattern was registered with the VS/BF medium. In contrast, the biomass cultured with BF concentrations showed high concentrations of lipoperoxides at the end of the first week of the experimental period (Figure 3A), reaching, for example, 2.8 µmol g^–1^ DT using BF 0.5%. However, these concentrations decreased significantly (*p* < 0.05) during the third week, mainly for the BF 0.5% and BF 1% treatments, reaching 1.3 and 1.2 µmol g^–1^ DT, respectively. The experimental factor that proved to be influential on the lipoperoxide concentrations, according to the Kruskal–Wallis test, was the treatment, i.e., culture medium utilized (*p* = 0.015). 

**Figure 4:**
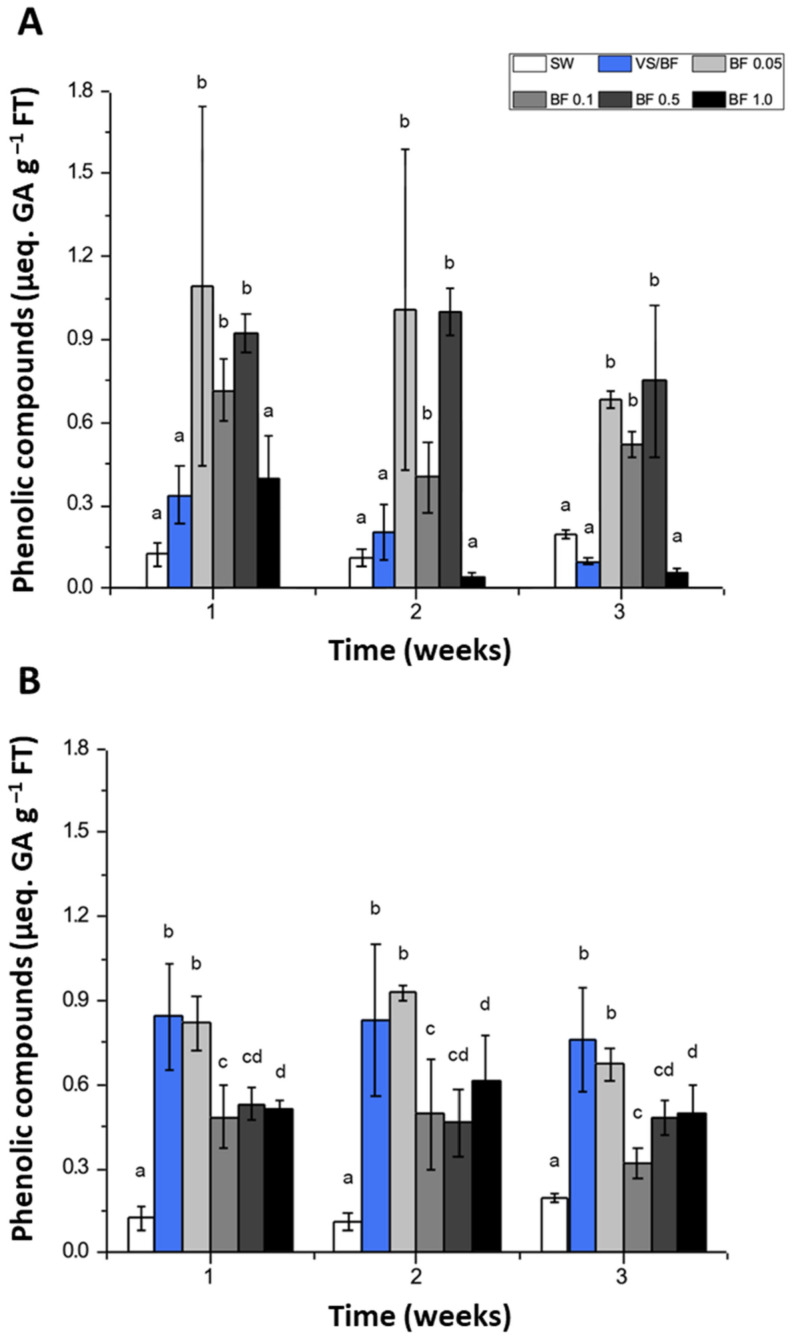
Phenolic compound concentration (µeq. GA g^–1^ FT, fresh tissue) in *G. chilensis* obtained under (**A**) indoor and (**B**) outdoor cultivation systems for each treatment evaluated (seawater (SW) treatment or control, Von Stosch/Basfoliar^®^ Aktiv (VS/BF) treatment, and Basfoliar^®^ Aktiv (0.05%, 0.1%, 0.5%, and 1% *v*/*v*) treatments) during the three weeks of the experimental period. Values are the mean ± SD of three replicates. The letters above the bar plots indicate the results of Dunn´s tests; means with the same letter are not significantly different at *p* ≥ 0.05.

Regarding the concentration of phenolic compounds determined in the control condition (SW treatment) under the indoor system, it showed a tendency to increase during the experimental period, although this was not significant (*p* > 0.05) (Figure 4A). The concentration at the end of the third week of the experimental period was 0.2 µeq. GA g^–1^ FT. In general, all treatments had a higher concentration of phenolic compounds than the SW treatment, except for the culture fertilized with BF 1.0% (lower than 0.2 µeq. GA g^–1^ FT) (Figure 4A). Finally, during the second and third weeks of the experimental period, the concentration of phenolic compounds principally using BF 1.0% showed a significant decrease (*p* < 0.05). The experimental factor that proved to be influential on the concentration of phenolic compounds, as obtained by the Kruskal–Wallis test, was the treatment (*p* < 0.05). In the outdoor cultivation system, the SW treatment showed lower concentrations for all weeks in the experimental period as compared with the other treatments (Figure 4B). The major concentrations were registered in the biomass cultured in VS/BF and BF 0.05% treatments (0.86 and 0.92 µeq. GA g^–1^ WT, respectively). 

**Figure 5:**
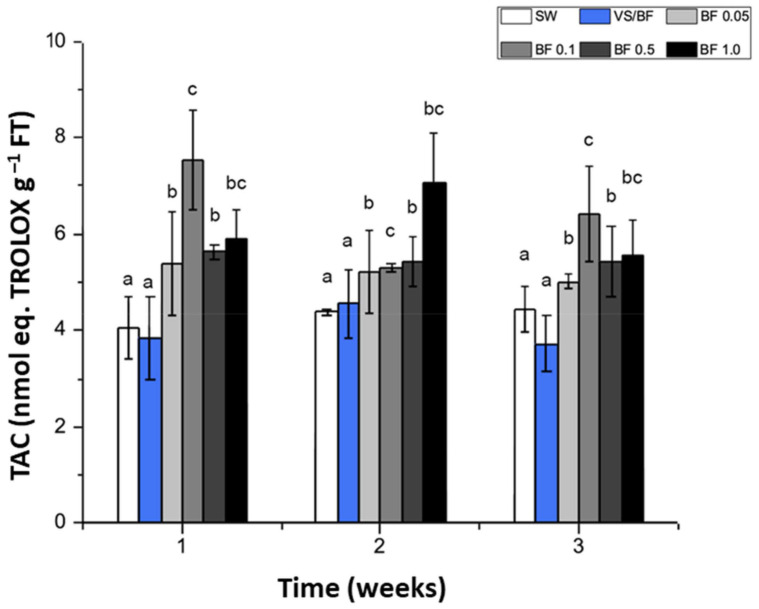
Total antioxidant capacity (TAC) (nmol eq. TROLOX g^–1^ FT, fresh tissue) in *G. chilensis* obtained under indoor cultivation for each treatment evaluated (seawater (SW) treatment or control, Von Stosch/Basfoliar^®^ Aktiv (VS/BF) treatment, and Basfoliar^®^ Aktiv (0.05%, 0.1%, 0.5%, and 1% *v*/*v*) treatments) during the three weeks of the experimental period. Values are the mean ± SD of three replicates. The letters above the bar plots indicate the results of Tukey’s test; means with the same letter are not significantly different at *p* ≥ 0.05.

The total antioxidant capacity (TAC) for *G. chilensis* was only determined under indoor conditions, according to the lower biomass stress and high concentration of phenolic compounds. The results showed that the TAC determined in the control, i.e., seawater (SW) treatment and the VS/BF treatment, were stable during all weeks of the experimental period, reaching ca 4.0 nmol eq. TROLOX g^–1^ FT (Figure 5). The major TAC was registered using BF. For example, during the first week of culture using BF 0.1%, the TAC was 7.5 nmol eq. TROLOX g^–1^ FT, and during the second week using BF 1%, the TAC was 7 nmol eq. TROLOX g^–1^ FT (Figure 5). No significant differences were registered along the culture time for each culture treatment (in all cases *p* > 0.05).

## 3. Discussion

In both types of cultivation system (indoor and outdoor), the enriched medium that showed the most promising results in terms of biomass and DGR was Basfoliar^®^ Aktiv (BF), as compared with the control treatment and the VS/BF medium. One of the explanations for this result may be the different compositions and concentrations of each medium. BF is a concentrate of macronutrients (N (NH_4_^+^), P (P_2_O_5_), and K (K_2_O)) and micronutrients (B, Cu, Fe, Mn, Mo, and Zn) [14]. The Von Stosch (VS) culture medium is a classic medium used in the initial steps of red seaweed cultivation [18,19], which contains N (NO_3_^–^), P (PO_4_^3–^), Fe, Mn, EDTA, and vitamins (B_1_, B_12_, and B_7–8_) [25]. Comparing the culture media, we found that BF had the higher content of N, P, and Fe than the VS medium, which could explain the better performance of the cultures under this treatment. Additionally, the chemical form of nitrogen can influence growth; many seaweed species have a higher affinity for ammonium given the different forms of uptake between nitrate (saturation kinetics) and ammonium (passive diffusion) [26]. Elements such as N and P are essential for algal growth, with N being the main growth-limiting element [27,28]. The contents of N and P depend on the bioavailability of each nutrient in the natural environment, which is directly related to seasonality and geographical location [27]. Fertilization with nitrogen increases N-based compounds such as amino acids, phycobiliproteins, and chlorophyll, thereby increasing the photosynthetic rate and growth, but reduces the content of phycocolloids such as agar and carrageenan [27]. It is also important to note that Fe has been shown to positively affect the growth of algal biomass [29,30], which could explain the results obtained in the present investigation. For example, under the BF concentration treatments, at the end of the third week of the experimental period, biomasses between 0.9 and 1.3 kg m^–2^ were determined in the indoor and outdoor cultivation systems, while under the SW (without fertilization step) treatment, a maximum biomass of 0.98 kg m^–2^ was obtained.

Nitrogen has been shown to be favorable for cultures of the *Gracilaria* spp. In terms of DGR [31,32], such as *G. tenuistipitata*, where a DGR of 3.1% was recorded in a pond culture with a concentration of 56 µg L^–1^ of N [31]. In our work, the maximum DGR values of 0.83 and 4.66% d^–1^ in the indoor and outdoor cultivation systems, respectively, were obtained with the BF concentration treatments at the end of the third week of the experimental period, and the minimum DGR values of –0.42% (indoor culture) and –0.10% d^–1^ (outdoor culture) were obtained with the VS/BF treatment at the same experimental time. Therefore, the N concentration and its chemical form in the BF stimulate the growth of *G. chilensis*. 

It has been shown that fertilization, or the supply of nutrients by pulses, is effective for cultures of *Gracilaria tikvahiae*, with the concentration of nutrients being more important than the frequency at which they are added [33]. In addition, it has been shown that the time required for exposure to fertilizers is relatively low in red algae of the genus *Gracilaria*, which double their N content within a few hours [34]. In algae, N is absorbed and stored during periods of high nutrient exposure, and then utilized in conditions of low bioavailability due to seasonal variations [35,36]. Thus, the methodology proposed for *G. chilensis* with fertilization enriched with N prior to the maintenance of the culture allowed high biomass development with excellent tissue quality.

Regarding biomass quality, it was found that lipoperoxide concentrations decreased during the experimental period, mainly under the BF concentration treatments at the end of the third week of the experimental period, which was related to the high concentration of phenolic compounds and high TAC under the same treatments. This observation was true principally for the indoor cultivation system. Environmental fluctuations, in terms of temperature, radiation, and photoperiod, influence the biomass growth and its quality. In fact, high levels of lipoperoxides have been reported to be related to the stress conditions to which the organisms are exposed [23,24,37,38,39]. Although several studies have reported that increased lipoperoxides are related to increased ROS production [23,40,41], the oxylipin-type lipoperoxides are related to cellular defense, since they act as signaling and/or protective compounds, such as antibacterial and wound-healing compounds in vegetables [42]. Therefore, caution should be exercised when high concentrations of lipoperoxides are measured, as they could be part of the oxylipins synthetized during the development of the culture’s conditions [23,42]. 

In terms of molecular markers, high concentration levels of phenolic compounds were identified in the cultures using Basfoliar^®^ Aktiv. However, these levels reduced as the experimental period progressed. This could be in line with study [23], where it was identified that the levels of phenolic compounds in organisms exposed to stress decreased due to being involved in stress tolerance mechanisms [23,24,43]. In addition, total antioxidant capacity (TAC) has been identified as an indicator of an organism’s ability to compensate for oxidative damage [43]. In this research, a high TAC was recorded in those cultures under Basfoliar^®^ Aktiv treatment, which indicated an adequate response to stress by *G. chilensis* under the cultivation conditions, and suggested an adequate biomass quality for obtaining molecules of interest. However, it is necessary to experiment with more than one type of antioxidant activity measurement to consider the different mechanisms of antioxidant action [44,45,46], and the identification of the responsible molecules.

Finally, the biomasses exposed to Basfoliar^®^ Aktiv (in particular, BF 0.05%) showed positive responses to stress that occurred during the cultivation of *G. chilensis*, given the high levels of phenolic compounds and TAC, concomitantly decreasing the levels of lipoperoxides, which confirmed the hypothesis that a fertilization step favors the cultivation of *G. chilensis*. Therefore, based on this research, it is suggested that the cultivation conditions applied in this study, using mainly BF as a source of nutrients, be applied for longer periods or even with intermittent nutrient pulsing (in stop-flow mode), as this would allow for high growth and adequate biomass quality.

## 4. Materials and Methods

### 4.1. Sampling

The initial vegetative biomass of *G. chilensis* (200 kg) was collected from Ancud, Chile (41°52′06″ S, 73°49′43″ W) and transported in cool conditions using ice packs to the Quintay Marine Research Center (CIMARQ). For the cultures, the methodologies proposed by the authors of [14,15] were used, which consisted of washing the biomass using tap water for 1 h. Then, the biomass was maintained and acclimatized for one week in outdoor raceways of 3000 L. In a constant flow of seawater filtered at 100 µm, covered with Raschel mesh with 80% light filtration, constant aeration, 32.3 PSU of salinity, and a light intensity between 150 and 230 µmol m^–2^ s^–1^. 

### 4.2. Experimental Setup

To evaluate biomass growth and quality, for both indoor and outdoor cultivation systems, VS/BF mix (VS/BF–A) [14] and Basfoliar^®^ Atkiv (0.05%, 0.1%, 0.5%, and 1% *v*/*v*) culture media were employed. The biomass was fertilized for 3 h weekly, utilizing the culture media mentioned above. A culture medium using only filtered seawater (100 µm) was used as the control (referred to as the control or SW treatment) (*n* = 3). The culture time was three weeks, and three independent replicates per culture medium were utilized. 

For the indoor cultivation system, 0.67 m^3^ cylindric white polypropylene tanks (*n* = 3) were used, where 5 kg of *G. chilensis* were placed in each of the tanks. The culture conditions were 32.3 PSU salinity, 16–18 °C, 12:12 light:dark (L:D) photoperiod, constant aeration, and a flow rate of 0.25 L s^–1^ of seawater filtered at 1 µm, with 0.5 m^3^ of final volume. The light intensity was gradually increased every week until 200–300 µmol m^–2^ s^–1^. A seawater volume of 0.1 m^3^ was considered for fertilization. Maintenance of the culture tanks was performed weekly, and tanks were cleaned with tap water. 

For the outdoor cultivation system, 0.4 m^3^ square polypropylene tanks (*n* = 3) were used with a 0.3 m^3^ final volume, and 3 kg of *G. chilensis* were placed in each of the tanks. The culture conditions were 32.3 PSU salinity, 16–18 °C, constant aeration, and a flow rate of 0.25 L s^–1^ of seawater filtered at 100 µm. To regulate light intensity, the tanks were covered with Raschel mesh with 80% light filtration. The light intensity was 120 µmol m^–2^ s^–1^, on average, and the photoperiod was according to the natural L:D fluctuation. A seawater volume of 0.1 m^3^ was considered for fertilization. Maintenance of the culture tanks was performed weekly, and tanks were cleaned with tap water.

The physicochemical parameters recorded throughout the experimental period in both types of cultivation systems were: oxidation–reduction potential (ORP, mV), conductivity (mS cm^–1^), turbidity (FNU), salinity (PSU), pH, dissolved oxygen (DO, mg L^–1^), and temperature (°C), using a HANNA^®^ Instruments multiparameter probe, model HI 9829. No significant variations during the entire experimental period were registered in both cultivation systems (in all cases *p* > 0.05) (Supplementary Materials, Table S1).

### 4.3. Determination of Biomass and Daily Growth Rate (DGR)

To determine the growth of *G. chilensis* in the indoor and outdoor cultivation systems, the biomass (kg m^–2^) was recorded every seven days using a digital scale and standardized with respect to the tank area considering the lower and lateral sections. The DGR calculation was based on study [47].

### 4.4. Determination of Biomass Quality Using Molecular Markers

Biomass was collected in triplicate from all cultures during each week of the experimental period to determine the quality of the biomass generated. This biomass was kept at –20 °C until further analysis. To determine the concentration of lipoperoxides, 2 g of *G. chilensis* biomass was dried in an oven at 40 °C until constant weight. The biomass was homogenized with liquid nitrogen in a ceramic mortar, and 5 mL of 0.1% *w*/*v* trichloroacetic acid (TCA) was added. The homogenate was centrifuged at 7400× *g* for 20 min. Lipoperoxides were detected by adding 100 µL of the clear homogenate to 900 µL of a reaction mixture containing 0.5% *w*/*v* thiobarbituric acid (TBA) in 20% *w*/*v* TCA, to a final volume of 1 mL [24]. The reaction mixture was incubated at 80 °C for 45 min, measuring the absorbance of the product at 512 nm in a spectrophotometer (Dynamica© model UV-VIS Ratio Beam Spectrophotometer Halo RB–10). To determine the amount of lipoperoxides, the molar absorptivity of the synthesized adduct (a = 155 mmol^–1^ cm^–1^) was used and expressed in µmoles g^–1^ DT (dry tissue).

To determine the concentration of phenolic compounds, 200 mg of fresh biomass of *G. chilensis* was used, which was homogenized with liquid nitrogen in a ceramic mortar; 2 mL of 0.1 M phosphate buffer, pH 7.0, was added during homogenization. The homogenate was centrifuged at 12,800× *g* for 10 min. The supernatant was recovered and stored at –20 °C. Then, 100 µL of the supernatant was used and mixed with 100 µL of 3% *w*/*v* sodium carbonate (Na_2_CO_3_), 650 µL of ultrapure water, and 150 µL of Folin–Ciocalteu reagent to a final volume of 1 mL. The reaction mixture was incubated for 2 h at room temperature, and the absorbance of the adduct was measured at 765 nm. A calibration curve was prepared with from 10 to 100 nmol mL^–1^ gallic acid. Total phenolic compounds were expressed as µeq. gallic acid (GA) g^–1^ FT (fresh tissue).

The total antioxidant capacity (TAC) was determined only in those cultures that obtained the highest biomass and concentration of phenolic compounds. For this purpose, a total Antioxidant Capacity Assay Kit, MAK 187 (Sigma-Aldrich, St. Louis, MO, USA) was used, where the Cu^2+^ ion was converted to Cu^+^ both by small molecules and proteins. The extraction was performed as described for the phenol’s extraction (see above). Then, 20 µL of supernatant per sample, 80 µL of ultrapure water, and 100 µL of the Cu^2+^ reagent mixture were used for a final volume of 200 µL per sample. The mixture was incubated for 90 min at room temperature, and the absorbance was measured at 570 nm in a multiplate reader (TECAN, model Infinite M Nano). Total antioxidant capacity was expressed as nmol eq. TROLOX g^–1^ FT, using a TROLOX standard curve (0–30 nmol L^–1^).

### 4.5. Data Analysis

Quantitative analyses were performed for the effect of experimental factors of interest (i.e., culture medium (VS/BF and Basfoliar^®^ Aktiv at different concentrations (0.05%, 0.1%, 0.5%, and 1%)) and culture time on biomass, DGR, and the molecular markers used to evaluate biomass quality (lipoperoxides, phenolic compounds, and TAC). To evaluate if there was a significant interaction between experimental factors, two-way repeated measures ANOVA or Kruskal–Wallis tests were performed, and this was followed by a Tukey’s test or Dunn´s test, respectively. All statistical analyses were performed in the statistical environment R package (R Development Core Team 2022), and significances were set at *p* < 0.05.

## 5. Conclusions

The results obtained in this research prove that indoor and outdoor cultivation and the use of enriched media (in a fertilization step), such as Basfoliar^®^ Aktiv, are beneficial principally in terms of *G. chilensis* biomass and quality. An indoor cultivation system can be more favorable than outdoor cultivation since it is possible to control a large number of culture parameters, in addition to supplying a high quantity of nutrients during fertilization, reducing the stress to which the organisms are exposed This allows high-quality biomass to be generated for use in the food, feed, pharmaceutical, and biotechnology industries.

## Data Availability

Derived data supporting the findings of this study are available from the corresponding author (L.C.-P.) on request.

## Supplementary Materials

The following supporting information can be downloaded at: https://www.mdpi.com/article/10.3390/plants12061340/s1, Table S1: Physicochemical parameters (pH, oxidation–reduction potential (ORP, mV), dissolved oxygen (DO, mg L^–1^), conductivity (mS cm^–1^), salinity (PSU), turbidity (FNU), and temperature (°C)) recorded during the cultivation of *G. chilensis* under the different treatments in both types of systems (indoor and outdoor cultivation).
