# Supplementary material for: Indoor and Outdoor Cultures of Gracilaria chilensis: Determination of Biomass Growth and Molecular Markers for Biomass Quality Evaluation
Source: Plants (Basel). 2023 Mar 16;12(6):1340. doi: 10.3390/plants12061340 (PMC10057914; doi:10.3390/plants12061340)
Supplement: Supplementary file 1 [file plants-12-01340-s001.zip › plants-2210666-supplementary.pdf]

plants

MDPI

Supplementary Material

Indoor and Outdoor Cultures of *Gracilaria chilensis*: Determination of Biomass Growth and Molecular Markers for Biomass Quality Evaluation

Sofía Caroca–Valencia[1,2,3,4], Jorge Rivas[1,2,3,4], Matías Araya[1,2,3,4], Alejandra Núñez[1,2,3,4], Florentina Piña[1,2,3,4,5], Fernanda Toro-Mellado[1,2,3,4,5] and Loretto Contreras–Porcia[1,2,3,4,*]

[1] Departamento de Ecología y Biodiversidad, Facultad de Ciencias de la Vida, Universidad Andres Bello, Santiago 8370251, Chile
[2] Centro de Investigación Marina Quintay (CIMARQ), Facultad de Ciencias de la Vida, Universidad Andres, Bello, Quintay, Valparaíso 2531015, Chile
[3] Center of Applied Ecology and Sustainability (CAPES), Santiago 8331150, Chile
[4] Instituto Milenio en Socio–Ecología Costera (SECOS), Santiago 8370251, Chile
[5] Programa de Doctorado en Biotecnología, Facultad de Ciencias de la Vida, Universidad Andres Bello, Santiago, Chile
* Correspondence: lorettocontreras@unab.cl

Table S1. Physicochemical parameters (pH, oxidation–reduction potential (ORP, mV), dissolved oxygen (DO, mg L^{-1}), conductivity (mS cm^{-1}), salinity (PSU), turbidity (FNU), and temperature (°C)) recorded during the cultivation of *G. chilensis* under the different treatments in both types of systems (indoor and outdoor cultivation).

Treatment	Week	pH	ORP	DO	mS cm^{-1}	PSU	FNU	°C
SW indoor	1	8	196	88	43	28	5	13
	2	8	193	87	42	27	6	13
	3	8	207	95	42	27	1	13
VS/BF indoor	1	8	195	95	43	28	22	13
	2	8	185	91	42	27	4	12
	3	8	215	94	42	27	23	14
BF 0.05% indoor	1	8	206	85	45	29	1	14
	2	8	187	87	45	29	1	15
	3	8	172	95	43	27	0	13
BF 0.1% indoor	1	8	221	89	39	25	1	15
	2	8	163	84	40	26	6	12
	3	8	216	79	40	26	4	12
BF 0.5% indoor	1	8	191	56	39	25	5	13
	2	8	185	72	39	25	6	14
	3	8	190	98	42	27	41	13
BF 1% indoor	1	8	177	100	39	25	49	13
	2	8	204	106	38	24	14	11
	3	8	217	103	39	25	28	14
SW outdoor	1	8	196	88	43	28	5	13
	2	8	193	87	42	27	6	13

	**3**	8	207	95	42	27	1	13
**VS/BF outdoor**	**1**	8	214	81	39	25	2	15
	**2**	8	179	86	41	26	0	12
	**3**	8	120	90	47	30	1	15
**BF 0.05% outdoor**	**1**	9	192	89	45	29	6	13
	**2**	8	176	88	45	29	1	14
	**3**	8	164	94	42	27	1	13
**BF 0.1% outdoor**	**1**	8	182	87	40	26	8	12
	**2**	8	213	84	39	25	2	15
	**3**	8	173	80	39	25	1	12
**BF 0.5% outdoor**	**1**	8	215	86	41	26	1	14
	**2**	8	170	90	42	27	9	14
	**3**	8	177	100	33	21	1	14
**BF 1% outdoor**	**1**	8	218	85	47	28	0	15
	**2**	8	175	93	44	27	2	12
	**3**	8	136	90	39	29	1	12
